# Model-based translation of results from *in vitro* to *in vivo* experiments for afabicin activity against *Staphylococcus aureus*

**DOI:** 10.1093/jac/dkae334

**Published:** 2024-09-24

**Authors:** Raphaël Saporta, Elisabet I Nielsen, Annick Menetrey, David R Cameron, Valérie Nicolas-Metral, Lena E Friberg

**Affiliations:** Department of Pharmacy, Uppsala University, Uppsala, Sweden; Department of Pharmacy, Uppsala University, Uppsala, Sweden; Translational Medicine Department, Debiopharm International SA, Lausanne, Switzerland; Translational Medicine Department, Debiopharm International SA, Lausanne, Switzerland; Translational Medicine Department, Debiopharm International SA, Lausanne, Switzerland; Department of Pharmacy, Uppsala University, Uppsala, Sweden

## Abstract

**Background:**

Translation of experimental data on antibiotic activity typically relies on pharmacokinetic/pharmacodynamic (PK/PD) indices. Model-based approaches, considering the full antibiotic killing time course, could be an alternative.

**Objectives:**

To develop a mechanism-based modelling framework to assess the *in vitro* and *in vivo* activity of the FabI inhibitor antibiotic afabicin, and explore the ability of a model built on *in vitro* data to predict *in vivo* outcome.

**Methods:**

A PK/PD model was built to describe bacterial counts from 162 static *in vitro* time–kill curves evaluating the effect of afabicin desphosphono, the active moiety of the prodrug afabicin, against 21 *Staphylococcus aureus* strains. Combined with a mouse PK model, outcomes of afabicin doses of 0.011–190 mg/kg q6h against nine *S. aureus* strains in a murine thigh infection model were predicted, and thereafter refined by estimating PD parameters.

**Results:**

A sigmoid E_max_ model, with EC_50_ scaled by the MIC described the afabicin desphosphono killing *in vitro*. This model predicted, without parameter re-estimation, the *in vivo* bacterial counts at 24 h within a ±1 log margin for most dosing groups. When parameters were allowed to be estimated, EC_50_ was 38%–45% lower *in vivo,* compared with *in vitro*, within the studied MIC range.

**Conclusions:**

The developed PK/PD model described the time course of afabicin activity across experimental conditions and bacterial strains. This model showed translational capacity as parameters estimated on *in vitro* time–kill data could well predict the *in vivo* outcome for a wide variety of doses in a mouse thigh infection model.

## Introduction

*Staphylococcus aureus* is an opportunistic human pathogen responsible for a wide range of infections, including acute bacterial skin and skin-structure infection (ABSSSI), endocarditis and bone and joint infection (BJI).^1–3^ MRSA-associated infections are particularly concerning.^4,5^ MRSA has been reported by the WHO as a high-priority pathogen for research and development of new antibiotics and by the CDC as a serious antibiotic resistance threat.^6,7^ Due to the emergence of antimicrobial resistance, new antibiotic classes with novel mechanisms of action are needed.

One therapeutic approach of interest is to impact the bacterial fatty acid biosynthesis II (FASII) pathway via inhibition of the enoyl-ACP reductase FabI.^8–10^ Afabicin is a prodrug of afabicin desphosphono, a FabI inhibitor targeting staphylococci.^11–15^ Afabicin is currently in Phase II development for staphylococcal BJI and ABSSSI.^16,17^

Understanding of the pharmacokinetics (PK) and pharmacodynamics (PD) for an antibiotic is one of the key components of the drug development process.^18,19^ Both *in vitro* and *in vivo* experimental models are commonly used to study the PK/PD relationship of antibiotics.^20–22^ The translation of preclinical information to suggest efficacious antibiotic doses in humans usually relies on PK/PD indices. These relate a drug exposure metric to a bacterial strain’s MIC. Bacterial load at a single point in time (usually 24 h after the start of treatment) is related to the PK/PD index values to derive PK/PD targets. However, this approach has limitations, highlighting the need for alternative translational approaches such as model-informed drug development (MIDD).^23–29^ PK/PD modelling has been used increasingly as a method to describe the time course of the antibiotic effect against bacteria.^30–32^ Previous work has shown the ability of a PK/PD model based on *in vitro* data to predict the PK/PD indices for different antibiotics,^29,33–35^ and model-based translation was recently explored.^36,37^

This study aimed to develop a model-based translation from *in vitro* time–kill data to *in vivo* mouse thigh infection data for afabicin against *S. aureus*, integrating available preclinical data generated during the drug development of afabicin. The ability to use a PK/PD model built from *in vitro* data to predict *in vivo* efficacy data was additionally explored, by comparing the model-predicted and observed bacterial response *in vivo*.

## Materials and methods

### Ethics

All animal studies were approved by Investigational Animal Care and Use Committees (IACUCs) at Eurofins Panlabs Taiwan and the Institute for Clinical Pharmacodynamics.

### *In vitro* time–kill experiments

Data were gathered from static *in vitro* time–kill experiments performed during afabicin drug development on 21 *S. aureus* strains, including 12 MRSAs, with afabicin desphosphono MICs ranging from 0.004 to 0.03 mg/L (Table S1, available as Supplementary data at *JAC* Online), which is reflective of the WT distribution for *S. aureus*.^38^ MICs were determined using broth microdilution according to CLSI guidelines (M07).^39^ The evaluated afabicin desphosphono concentrations ranged from 0.004 to 1 mg/L, and all experiments included growth controls without drug. Briefly, bacteria from fresh overnight agar cultures were incubated in Mueller–Hinton Broth II (MHBII) at 35°C with shaking to reach the log-growth phase. The suspensions were diluted to reach target bacterial concentrations ranging from 1.6 × 10^5^ to 2.0 × 10^7^ cfu/mL. At time zero, 24-well plates (24 curves), 15 mL tubes (44 curves) or 125 mL Erlenmeyer flasks (94 curves) were loaded with MHBII broth, afabicin desphosphono diluted to achieve target concentration and the inoculum suspension, for a final volume of either 2, 10 or 20 mL. Vessels were incubated at 35°C, and 10–50 µL samples were taken at multiple times up to 24 or 48 h for viable count determinations. Samples were diluted in 10-fold series, plated and incubated for 16 to 24 h. The cfu were counted manually from a total of 162 time–kill curves, generating 722 cfu counts (including 8 below the limit of detection of 200 cfu/mL) that were available for model development.

### *In vivo* mouse efficacy studies

The *in vivo* efficacy studies were performed during the development of afabicin, using a neutropenic mouse thigh infection model. Female ICR or Swiss Webster mice (*n* = 952) were rendered neutropenic by intraperitoneal (IP) injections of cyclophosphamide at 150 mg/kg on Day 4 and 100 mg/kg on Day 1 before infection. On Day 0, mice were infected with an intramuscular injection of an inoculum ranging from 8.0 × 10^4^ to 1.3 × 10^7^ cfu, selected based on the strains’ ability to grow in controls, in the left thigh. Nine *S. aureus* strains, including six MRSAs, with afabicin desphosphono MIC ranging from 0.004 to 0.06 mg/L, were studied (Table S1), including one strain that was also studied *in vitro* (*S. aureus* ATCC 29213). For mice not belonging to the control groups, afabicin treatment was initiated 2 h after infection. Afabicin single doses of 129 mg/kg or doses ranging from 0.011 to 190 mg/kg every 6 h were administered IV (*n* = 82) or IP (*n* = 430). Mice were euthanized at 2, 26, 50 or 74 h after infection. Thighs were removed aseptically, homogenized, diluted in 10-fold dilution steps, and plated on agar. The cfu were counted after 20 to 24 h of incubation at 35°C.

### PK/PD model for *in vitro* data

A PK/PD model was developed based on the data from the time–kill experiments. In the model structure, two possible bacterial states were assumed: growing and afabicin desphosphono-susceptible bacteria (S), and dormant (or resting), non-growing and non-susceptible bacteria (D), with bacteria from both states expected to grow after dilution during the cfu counting procedure.^40^ Model development was initiated using the strain with the richest data (*S. aureus* ATCC 29213), and thereafter using data from strain ATCC 43300, and finally models were fit to cfu data from all strains. The growth control experiments were first analysed alone to estimate bacteria-related parameters, i.e. the bacterial growth rate (k_growth_), the bacterial natural death rate (k_death_) and the maximum bacterial count in the system (B_max_). These parameter estimates were then fixed and drug-related parameters were estimated on all time–kill experiments. All bacteria were assumed to be in the S state at the start of the experiments; however, the possibility that part of the bacterial population could be in the D state at the start of the experiment was also evaluated. The afabicin desphosphono effect was included as an additional killing rate constant (k_drug_) for the S bacteria. The bacterial system was described by the following equations:


(1) $$\begin{array}{c} \frac{\mathrm{dS}}{\mathrm{dt}}=k_{\mathrm{growth}}\;\times \;S\;\text{–}\;(k_{\mathrm{death}}+k_{\mathrm{drug}})\;\times \;S\;\text{–}\;k_{\mathrm{SD}}\;\times \;S\; \end{array}$$

(2) $$\begin{array}{c} \frac{\mathrm{dD}}{\mathrm{dt}}=k_{\mathrm{SD}}\;\times \;S\;\text{–}\;k_{\mathrm{death}}\;\times \;D\; \end{array}$$

The transfer rate from the S to D state (k_SD_) was defined as k_SD _= (S + D)×(k_growth−_k_death_)/B_max_.

The afabicin desphosphono effect (k_drug_) was evaluated by linear, power, E_max_ or sigmoid E_max_ functions. A delay in effect was explored through either the addition of an effect compartment for afabicin desphosphono concentrations, a steep increase (i.e. ‘turn on’) of k_drug_ at an estimated timepoint, or an additional bacterial lag state. To describe observed regrowth at the end of experiments, compartments representing a reversible adaptation of the bacteria to afabicin desphosphono were evaluated.^41^ The compartments represent fractions of no adaptation (AS_off_), or reduced susceptibility due to adaptation (AS_on_), with the entire fraction being in AS_off_ at the start of the experiments (AS_off _= 1, AS_on _= 0 at start of experiment). In the presence of afabicin desphosphono, adaptation was initiated, which can transfer back to the no-adaptation state as described in the following equations:


(3) $$\begin{array}{c} \frac{\mathrm{dA}S_{\mathrm{off}}}{\mathrm{dt}}=k_{\mathrm{off}}\;\times \;AS_{\mathrm{on}}\;\text{–}\;k_{\mathrm{on}}\times \;C\;\times \;AS_{\mathrm{off}} \end{array}$$

(4) $$\begin{array}{c} \frac{\mathrm{dA}S_{\mathrm{on}}}{\mathrm{dt}}=k_{\mathrm{on}}\;\times \;C\;\times \;AS_{\mathrm{off}}\;\text{–}\;k_{\mathrm{off}}\;\times \;AS_{\mathrm{on}} \end{array}$$

Adaptation was activated through the rate constant k_on_, dependent on the afabicin desphosphono concentration C, and reversed through the rate constant k_off_. The available data could not support estimation of the rate of reversal of adaptation, and therefore k_off_ was fixed to 0.0139 h^−1^ to represent a half-life of 50 h.^41^ Adaptation was implemented as a reduction of the drug effect, e.g. an increase in EC_50_ or a reduction of the E_max_ parameter (with k_drug_ described by a sigmoid E_max_ function) so that:


(5) $$\begin{array}{c} E_{\max}(t)=E_{\max}\;\times \;\left(1\;-\;\frac{AS_{\mathrm{on}}(t)}{AS_{\mathrm{on}}(t)+AS_{50}}\right) \end{array}$$

where AS_50_ represents the fraction in AS_on_ when E_max_ is reduced by 50%.

In order to account for strain differences in afabicin desphosphono effect, scaling of the EC_50_ (with k_drug_ described by a sigmoid E_max_ function) by the strains’ MIC values was tested with a one (β or γ fixed to 1) or two (β and γ estimated) parameter implementation:^42^


(6) $$\begin{array}{c} EC_{50}=\beta \;\times \;\mathrm{MI}C^{\gamma } \end{array}$$

### PK/PD model for *in vivo* data

The final PK/PD model based on *in vitro* data was used as the model structure for the *in vivo* data. Due to the limited number of measurement times in the mouse efficacy studies, both the *in vitro* and *in vivo* data were analysed simultaneously. In a first step, the bacteria-related parameters were re-estimated using growth control data, with k_growth_ and k_death_ being shared between *in vitro* and *in vivo*, and B_max_ differing between *in vitro* and *in vivo*. The re-estimated parameters were fixed in the remaining analysis.

A mouse PK model for afabicin was used to derive the afabicin desphosphono concentration−time profiles for the mouse efficacy studies.^43^ All mice were assumed to have the typical PK parameters, which were assumed to be the same following IV or IP afabicin administrations. The unbound (*f*_u_ = 0.02) afabicin desphosphono concentration in the central compartment was driving the effect in the PK/PD model. No effect of the prodrug afabicin on bacterial killing was considered in the PK/PD model.

A stepwise approach was used to re-estimate the parameters describing the afabicin desphosphono effect.^37^ Parameters were initialized to their final value from the PK/PD model based on *in vitro* data. Two distinct residual variability parameters were estimated for *in vitro* and *in vivo* data. Ratios were added to each estimated effect parameter, so that $\mathrm{Para}m_{\mathrm{vivo}}=\mathrm{Para}m_{\mathrm{vitro}}\times \mathrm{Rati}o_{\mathrm{Param}}$. By default, these ratios were fixed to 1, implying no difference between the *in vitro* and *in vivo* parameter estimates. In the forward inclusion steps, ratios were unfixed one by one and the parameters and unfixed ratios were estimated. The model with the most significant improvement in objective function value (OFV) was kept, and the inclusion process was repeated by unfixing additional ratios one by one until no significant improvement in OFV was obtained. In backward steps, starting from the final model of the forward steps, ratios were fixed back one by one to a value of 1, and parameters were estimated. The model with the lowest non-significant worsening in OFV was kept, and the backward step was repeated until fixing any of the remaining ratios led to a significant OFV worsening.

### Prediction of in vivo efficacy from the PK/PD model based on *in vitro* data

The ability of the developed PK/PD model based on *in vitro* data to predict the *in vivo* outcome was evaluated. k_growth_ and B_max_ were fixed to their *in vivo* estimates from the PK/PD modelling of *in vivo* data. Based on the uncertainty of the *in vitro* estimates, 500 sets of afabicin desphosphono effect parameters were sampled, and the parameters were used to predict the bacterial count over time using the design of the *in vivo* efficacy studies. The bacterial counts predicted by the models were compared with the observed bacterial counts in the efficacy studies.

### Data analysis and software

Model development was performed in NONMEM version 7.5.0^44^ and Perl-speaks-NONMEM (PsN) version 5.2.6,^45^ using the first-order conditional estimation with interaction method. R version 4.2.1 and the xpose4 package were used for data management and graphical analysis.^46,47^ In the PK/PD modelling, a log_10_-transform-both-side approach was used, with a log_10_-transformed additive error model. Interindividual variability of the parameters was not explored. Model selection was driven by scientific plausibility, the difference in OFV (ΔOFV), where a difference of 10.83 (*P* < 0.001) was required for inclusion of one parameter, parameter precision and graphical analysis,^48^ including visual predictive checks (VPCs),^49^ and prediction-corrected VPCs (pcVPCs).^50^ Simulations were performed in NONMEM and in R using mrgsolve version 1.0.6.^51^

## Results

### PK/PD model for *in vitro* data

**Figure 1.**
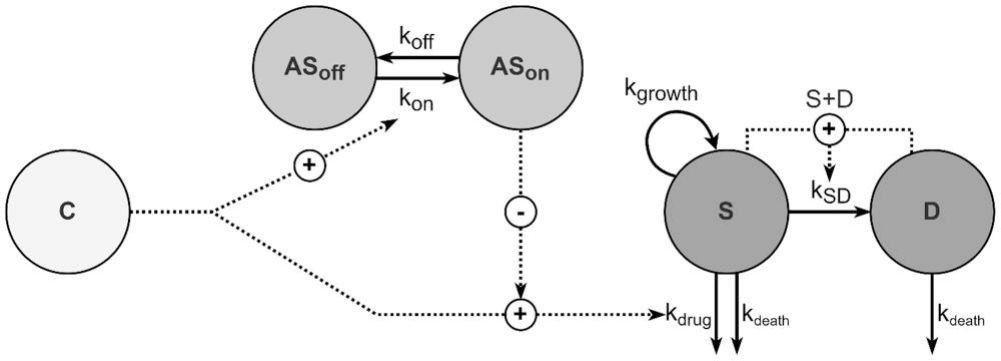
Schematic representation of the PK/PD model. AS_off_, no adaptive susceptibility; AS_on_, adaptive susceptibility; C, afabicin desphosphono unbound concentration; k_death_, bacteria natural death rate constant; k_drug_, afabicin desphosphono effect rate constant; k_growth_, bacteria growth rate constant; k_off_, adaptation offset rate constant; k_on_, adaptation onset rate constant; k_SD_, transfer to dormant state rate constant; D, dormant bacteria; S, susceptible bacteria.

**Figure 2.**
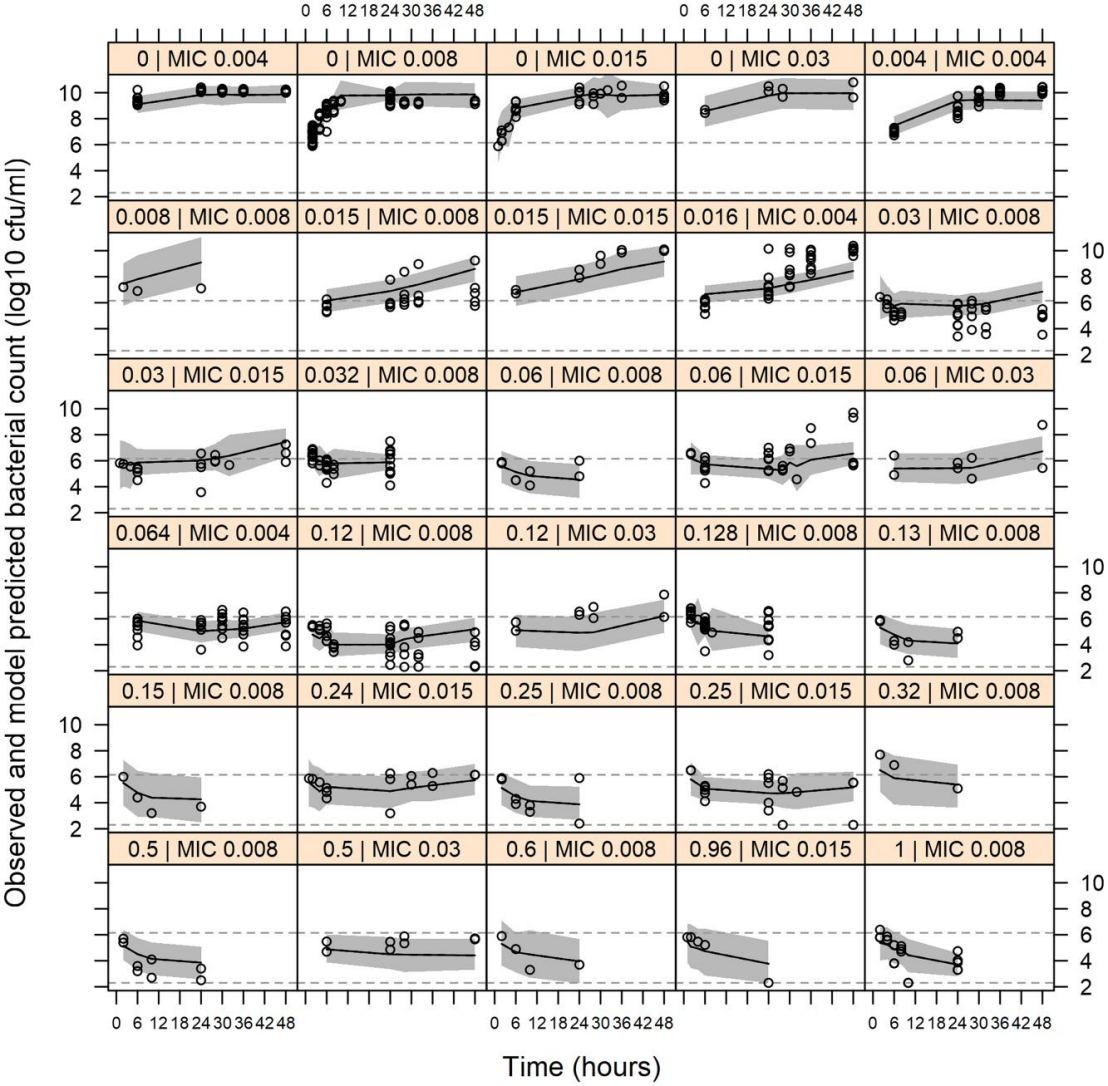
VPCs of the PK/PD model for *in vitro* data. Each panel presents time–kill data of one afabicin desphosphono concentration (left value, mg/L) for all strains sharing a given MIC (right value, mg/L). Shown are the observed bacterial counts (open circles), the median simulated bacterial counts (solid lines) and the 95% CIs for the simulated medians (grey shaded area). Grey broken lines are the median starting inocula from all experiments (top line) and the limit of detection (bottom line). This figure appears in colour in the online version of *JAC* and in black and white in the print version of *JAC*.

The structure of the developed PK/PD model is presented in Figure 1. Bacterial parameters k_growth_ and B_max_ could be estimated from the growth control data, whereas k_death_ was not significantly different from the literature value of 0.179 h^−1^ (ΔOFV = 0.99) and was therefore fixed to the literature value for simplicity and consistency with previous studies.^40^ Afabicin desphosphono effect was implemented as a sigmoid E_max_ function, with adaptation compartments for a reduction of E_max_. The AS_50_ parameter was estimated to be 0.861, corresponding to a reduction of E_max_ of ∼54% at maximal adaptation. Differences in drug effect between the *S. aureus* strains were estimated with a one-parameter implementation of MIC on EC_50_ (ΔOFV = 30.7), with β fixed to 1 and γ being estimated as 0.16, representing a ∼12% higher EC_50_ when MIC is doubled. All parameters were estimated with acceptable precision (<40%, Table 1). VPCs displayed a good fit of the model across all strains and afabicin desphosphono concentrations (Figure 2).

**Table 1.** Parameter estimates and relative standard errors (RSEs) of the PK/PD models based on *in vitro* data alone, and *in vitro* and *in vivo* data

			PK/PD model based on *in vitro* data		PK/PD model based on *in vitro* and *in vivo* data	
Parameter	Description	Units	Estimate	(RSE, %)	Estimate	(RSE, %)
k_growth_	Bacterial growth rate constant	h^−1^	1.13	(2.0^a^)	1.10	(2.0^a^)
k_death_	Bacterial natural death rate constant	h^−1^	0.179	(Fixed)	0.179	(Fixed)
B_max vitro_	Maximum system capacity—*in vitro*	log_10_ cfu/mL	9.85	(1.0^a^)	9.86	(1.0^a^)
B_max vivo_	Maximum system capacity—*in vivo*	log_10_ cfu/g	—	—	9.08	(1.0^a^)
E_max_	Maximum afabicin desphosphono killing rate constant	h^−1^	4.10	(7.0)	3.37	(7.2)
β_vitro_	EC_50_ scaling slope—*in vitro*	—	1	(Fixed)	1	(Fixed)
β_vivo_	EC_50_ scaling slope—*in vivo*	—	—	—	0.707	(6.0)
γ	EC_50_ scaling power	—	0.160	(36)	0.204	(21)
Hill	E_max_ model sigmoidicity factor	—	0.360	(6.4)	0.324	(6.3)
k_on_	Adaptation onset rate constant	mL/(µg·h)	0.406	(24)	0.386	(19)
k_off_	Adaptation offset rate constant	h^−1^	0.0139	(Fixed)	0.0139	(Fixed)
AS_50_	Fraction needed to reach 50% reduction in E_max_	—	0.861	(13)	1.18	(12)
RES_vitro_^b^	Residual variability—*in vitro*	log_10_ cfu/mL	0.869	(5.0)	0.877	(5.1)
RES_vivo_^b^	Residual variability—*in vivo*	log_10_ cfu/g	—	—	0.621	(4.1)

^a^These parameters were fixed based on estimates from a model with growth control data only.

^b^RES parameters correspond to the magnitude of residual unexplained variability on the standard deviation scale.

### Prediction of *in vivo* efficacy from the PK/PD model based on *in vitro* data

**Figure 3.**
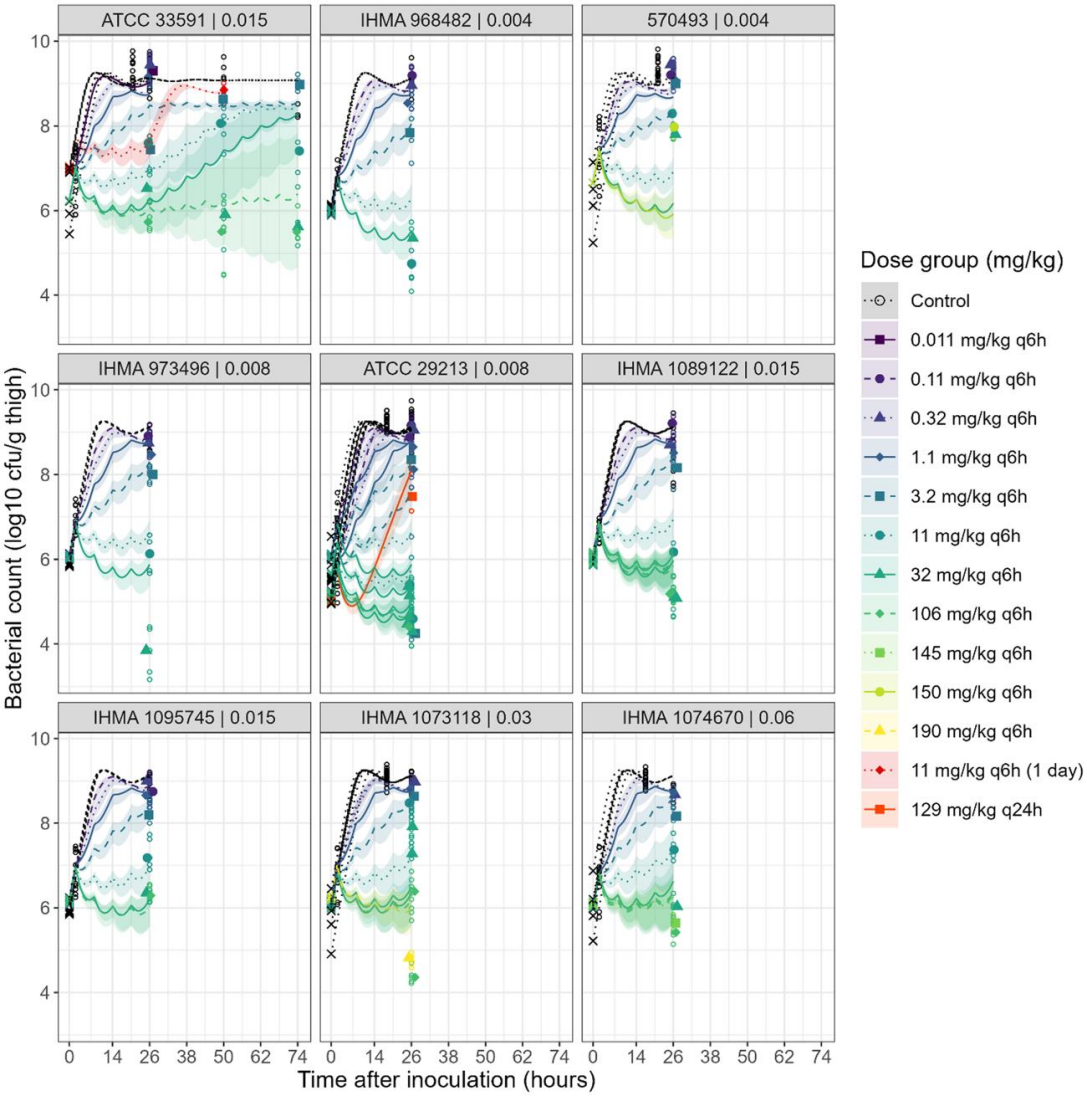
Predictions of *in vivo* efficacy based on the PK/PD model for *in vitro* data, accounting for parameter uncertainty. Shown are the starting inocula (crosses), observed bacterial counts (open circles), the median observed bacterial counts for treated groups (solid symbols) and the median (lines) and 95% CI (shaded areas) of predictions from models with parameter estimates sampled in the uncertainty distribution for the different afabicin dosing regimens and strains. Panels present data for each *S. aureus* strain (strain ID | MIC in mg/L). Study groups with similar starting inoculum for a same strain and dose were grouped in the plots.

The *in vivo* bacterial time profiles predicted from *in vivo* specific growth parameters and the PK/PD model based on *in vitro* data, with intervals for predictions considering parameter uncertainty, are presented in Figure 3 and Figure S1. The model was able to predict most of the *in vivo* data, with the median of predictions within a 1 log_10_ margin of most observations. Observed counts were notably underpredicted for most treatment groups of *S. aureus* 570493 and the 11 and 32 mg/kg q6h groups for *S aureus* IHMA 1073118, and overpredicted for the highest dose group (190 mg/kg q6h). Furthermore, the model predicted regrowth at 48 and 72 h in the 32 mg/kg q6h group for ATCC 33591, which was not observed *in vivo*. However, when considering parameter uncertainty, bacterial counts were well predicted at 24 h, and at 48/72 h for the lower afabicin doses. At 48 and 72 h for the higher afabicin doses, the intervals were too wide, and consequently an evaluation was not feasible.

### PK/PD model for *in vivo* data

**Figure 4.**
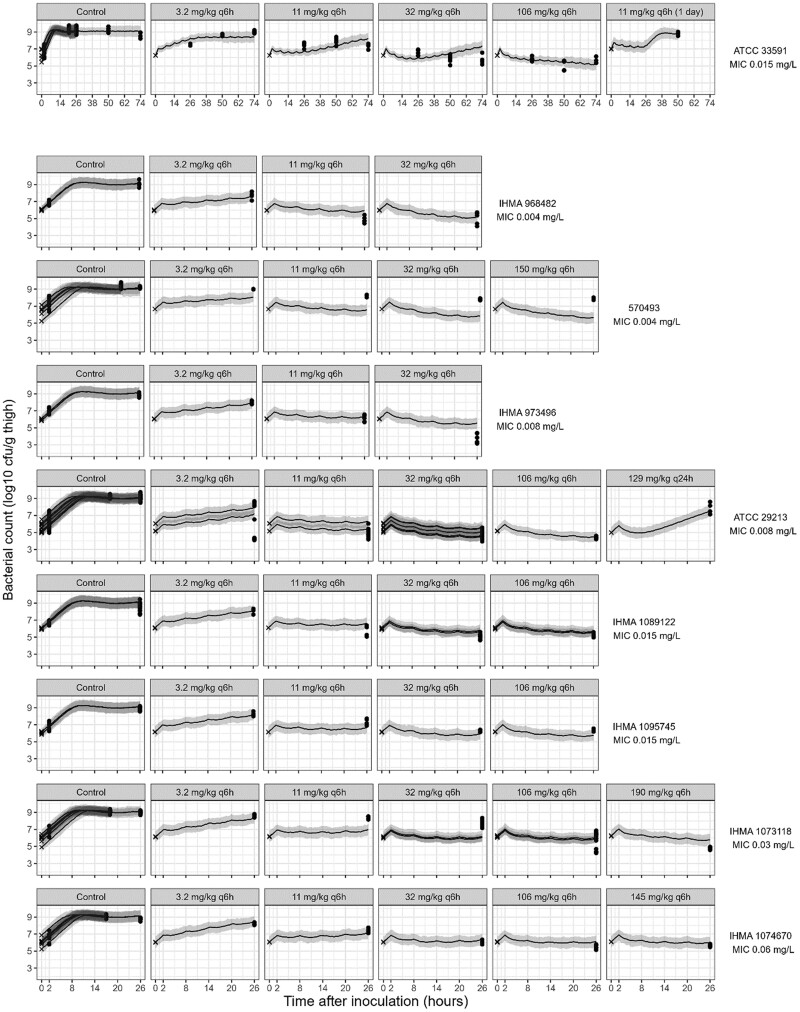
VPCs of the PK/PD model for *in vivo* data. Shown are the starting inocula (crosses), observed bacterial counts (solid circles), with the medians (lines) and the corresponding 95% CIs of the median (shaded areas) based on model predictions for the different afabicin dosing regimens and strains. Each row presents panels containing data for the different dose groups for a given strain. Panels for doses below 3.2 mg/kg are presented in Figure S2.

When re-estimated on both the *in vitro* and *in vivo* data, k_growth_ was slightly lower (1.10 versus 1.13 h^−1^), while the *in vivo* B_max_ was estimated as 9.08 log_10_ cfu/g thigh. In the final model (Table 1), only the EC_50_ scaling slope β was estimated as having a specific *in vivo* value, with adaptation and all other parameters remaining shared (ΔOFV = 121 compared with a model with all parameters shared). The *in vivo* EC_50_ values estimated with the PK/PD model were 38% to 45% lower within the studied MIC range in comparison with the estimates from the PK/PD model for *in vitro* data (e.g. for an MIC of 0.008 mg/L, the estimated *in vivo* EC_50_ was 0.26 mg/L, versus 0.46 mg/L for *in vitro* data; for an MIC of 0.03 mg/L, *in vivo* EC_50_ of 0.35 mg/L versus 0.57 mg/L in the PK/PD model for *in vitro* data). Due to limited availability of *in vivo* data in the first hours, k_on_ and AS_50_ parameters had to be shared between *in vitro* and *in vivo*. This model performed better than a model assuming no adaptation *in vivo* (ΔOFV = 112.7). The re-estimated parameters resulted in acceptable precision (<30%, Table 1). Other efficacy parameters were similar to their estimates from the PK/PD model for *in vitro* data. At maximal adaptation, k_drug_ was estimated as ∼1.82 h^−1^*in vivo*, compared with an estimate of ∼1.89 h^−1^ in the PK/PD model based on *in vitro* data. VPCs suggested a good ability of the re-estimated model to describe the *in vivo* data for the different strains (Figure 4 and Figure S2). Counts were systematically underpredicted for treatment groups of *S. aureus* 570493, where bacterial growth was observed. Model fit for *in vitro* data was only slightly worsened with the re-estimated model (increase in OFV of 12.06 for *in vitro* data).

## Discussion

In this study, a PK/PD modelling framework was developed to describe the effect of afabicin desphosphono over time, both *in vitro* in the setting of time–kill experiments, and *in vivo* in a mouse thigh infection model.

The PK/PD model for *in vitro* data was able to describe 48 h time–kill data from 21 *S. aureus* strains with different MICs. Bacteria were described to enter dormancy when approaching the stationary growth phase, due to less favourable environmental conditions such as lack of space and starvation.^52^ The model used only one set of parameters, with MIC describing differences in drug effect between strains by scaling the EC_50_ parameter. This is consistent with previous studies for other antibiotics, where a correlation between MIC and EC_50_ has been observed.^42,53–56^ Differences in MIC were, however, associated with a moderate increase in EC_50_ (e.g. estimated *in vivo* EC_50_ of 0.23 and 0.26 mg/L for MICs of 0.004 and 0.008 mg/L, respectively). EC_50_ is expected to describe drug effect across bacterial loads, whereas MIC is a marker of observed efficacy at high bacterial counts, which originates from a single assessment of visible growth, affected by strain characteristics like the growth rate, with known limitations.^57^ The adaptation compartments allowed description of the slower decrease in bacterial count or regrowth observed at the later timepoints in the time–kill experiments. PK/PD models have earlier shown the ability to predict bacterial dynamics across strains and inoculum sizes.^58^ The parameters in the PK/PD model for *in vitro* data were not specific to one given strain, which facilitated predictions for strains and MICs that were not present in the data used to develop the model.

The *in vivo* efficacy studies evaluated the effect of afabicin against nine *S. aureus* strains, with MICs of a wider range than what was available *in vitro*. Furthermore, *in vitro* time–kill data were available for only one of these strains, i.e. for eight out of nine strains, PK/PD model predictions were both a translation from *in vitro* to *in vivo*, as well as a translation across strains. Despite these differences, the PK/PD model based on *in vitro* data showed a good ability to predict the *in vivo* outcome when only assuming knowledge of afabicin mouse PK and of the *in vivo* k_growth_ and B_max_. As the latter parameters are related to the bacterial strains and the infection model, they were estimated based on growth control experiments alone. Growth control data can be used across experiments with various compounds and thereby allow predictions to be performed before generating new growth control data. This exemplifies how a model-based approach could be used to predict outcomes and inform dose selection for *in vivo* efficacy studies before any *in vivo* efficacy study has been performed.

The main difference estimated between the PK/PD model based on *in vitro* data alone and the PK/PD model that was re-estimated on the *in vitro* and *in vivo* data was a lower *in vivo* EC_50_ for afabicin desphosphono. One explanation for this difference could be that the unbound concentration at the infection site is higher than expected, possibly linked to the good tissue distribution properties of afabicin desphosphono.^59^ These properties have been observed in humans, where a previous study of afabicin desphosphono concentrations in bone tissues in patients indicated good penetration properties, notably with a mean unbound AUC ratio of synovial fluid over plasma of 2.88.^60^ Another explanation for this lower EC_50_*in vivo* could be high concentrations of afabicin desphosphono in the phagocytic cells remaining after neutropenia induction, as the ability of afabicin desphosphono to accumulate in macrophages while remaining soluble, free and active against intracellular *S. aureus* has been previously described.^61^ Other parameters were estimated with values similar to their *in vitro* estimates. The re-estimation step significantly improved the model fit compared with the use of *in vitro* parameters alone.

In addition to the integration of multiple types of data within one modelling framework, one strength of this study was the availability of 48 and 72 h data, which allowed us to describe and make predictions after 24 h, the timepoint when animal studies usually end. Another strength was the availability of a large amount of cfu data from 29 *S. aureus* strains with varying MICs. The ability of model-based translation to predict efficacy in strains that were not studied before was also evaluated.

One weakness in the analysis was the single timepoint nature of the *in vivo* data, although this is common for bacterial infection studies in mice. This did not permit us to re-evaluate the model structure or estimate the *in vivo* model parameters without relying on the *in vitro* data. Notably, reduced effects of afabicin due to adaptation and/or increased MIC were not routinely assessed in *in vivo* studies. Additionally, although strains with different MICs were studied, all studied strains were susceptible to afabicin desphosphono. The PK model used in this analysis was based on data from female ICR mice. Whilst most of the animal studies used female ICR mice (*n* = 568), Swiss Webster mice were also used (*n* = 384). Potential differences in PK between the two mouse types cannot be excluded.

In conclusion, a model-based approach was established to describe afabicin activity while integrating both *in vitro* and *in vivo* data. The developed PK/PD models consider the full time course of PK and efficacy, unlike the PK/PD index approach that considers summary metrics of PK and one efficacy timepoint. Furthermore, a PK/PD model developed based on *in vitro* data alone demonstrated the ability to predict the outcome of *in vivo* neutropenic mouse thigh infection studies. The model-based approach allows simulations of a wide range of experimental conditions to explore various scenarios, with iterative model refinement as more data become available. In the drug development setting, a model-based approach could be used to help dose selection in animal dose fractionation studies using the available *in vitro* data. In a next step, the model could be used in combination with PK data from patients to predict an efficacious dosing regimen based on simulations of bacterial dynamics, which could be compared with the prediction from the standard PK/PD index approach.

## Supplementary Material

dkae334_Supplementary_Data

## Supplementary data

Figures S1 and S2 and Table S1 are available as Supplementary data at *JAC* Online.
