# Supplementary material for: Model-based translation of results from in vitro to in vivo experiments for afabicin activity against Staphylococcus aureus
Source: J Antimicrob Chemother. 2024 Sep 24;79(12):3150–9. doi: 10.1093/jac/dkae334 (PMC11638087; doi:10.1093/jac/dkae334)
Supplement: dkae334_Supplementary_Data [file dkae334_supplementary_data.docx]

**Table S1.** List of *Staphylococcus aureus* strains used in this study

| Experiment setting | Strain name | Relevant Resistance phenotype | Afabicin desphosphono MIC (mg/L)^1^ |
| --- | --- | --- | --- |
| *In vitro* | 772 | MRSA | 0.015 |
| *In vitro* | 779 | MSSA | 0.008 |
| *In vitro* | 1008 | MSSA | 0.008 |
| *In vitro* | 1137 | MRSA | 0.008 |
| *In vitro* | 1654 | MSSA | 0.008 |
| *In vitro* | 1659 | MRSA | 0.008 |
| *In vitro* | 1725 | LRSA | 0.008 |
| *In vitro* | 2012 | VISA | 0.008 |
| *In vitro* | 2153 | MRSA | 0.015 |
| *In vitro* | 2293 | MRSA | 0.008 |
| *In vitro, in vivo* | ATCC 29213 | MSSA | 0.004 - 0.008 |
| *In vitro* | ATCC 43300 | MRSA | 0.004 - 0.015 |
| *In vitro* | D0211784 | MRSA | 0.008 |
| *In vitro* | IHMA 983301 | MRSA | 0.004 - 0.008 |
| *In vitro* | IHMA 983305 | MRSA | 0.004 - 0.008 |
| *In vitro* | IHMA 1073667 | MSSA | 0.004 - 0.015 |
| *In vitro* | IHMA 1087759 | MRSA | 0.015 - 0.03 |
| *In vitro* | IHMA 1099486 | MRSA | 0.004 - 0.008 |
| *In vitro* | IHMA 1146939 | MSSA | 0.004 - 0.015 |
| *In vitro* | IHMA 1158923 | MRSA | 0.015 - 0.03 |
| *In vitro* | IHMA 1188641 | MSSA | 0.004 - 0.015 |
| *In vivo* | ATCC 33591 | MRSA | 0.015 |
| *In vivo* | IHMA 1089122 | MRSA | 0.015 |
| *In vivo* | IHMA 1073118 | MRSA | 0.03 |
| *In vivo* | IHMA 968482 | MRSA | 0.004 |
| *In vivo* | IHMA 973496 | MRSA | 0.008 |
| *In vivo* | IHMA 1095745 | MSSA | 0.015 |
| *In vivo* | IHMA 1074670 | MRSA | 0.06 |
| *In vivo* | 570-493 | MSSA | 0.004 |

^1^MIC values used in the PKPD model were based on MIC determination assays performed during the time-kill experiments when available. MIC value ranges are reported when different MIC values were obtained in different experiments.

Abbreviations: ATCC, American Type Culture Collection; IHMA, International Health Management Associates; LRSA, linezolid-resistant *S. aureus*; MIC, minimum inhibitory concentration; MRSA, methicillin-resistant *S. aureus*; MSSA, methicillin-susceptible *S. aureus*; Vancomycin-intermediate S. aureus


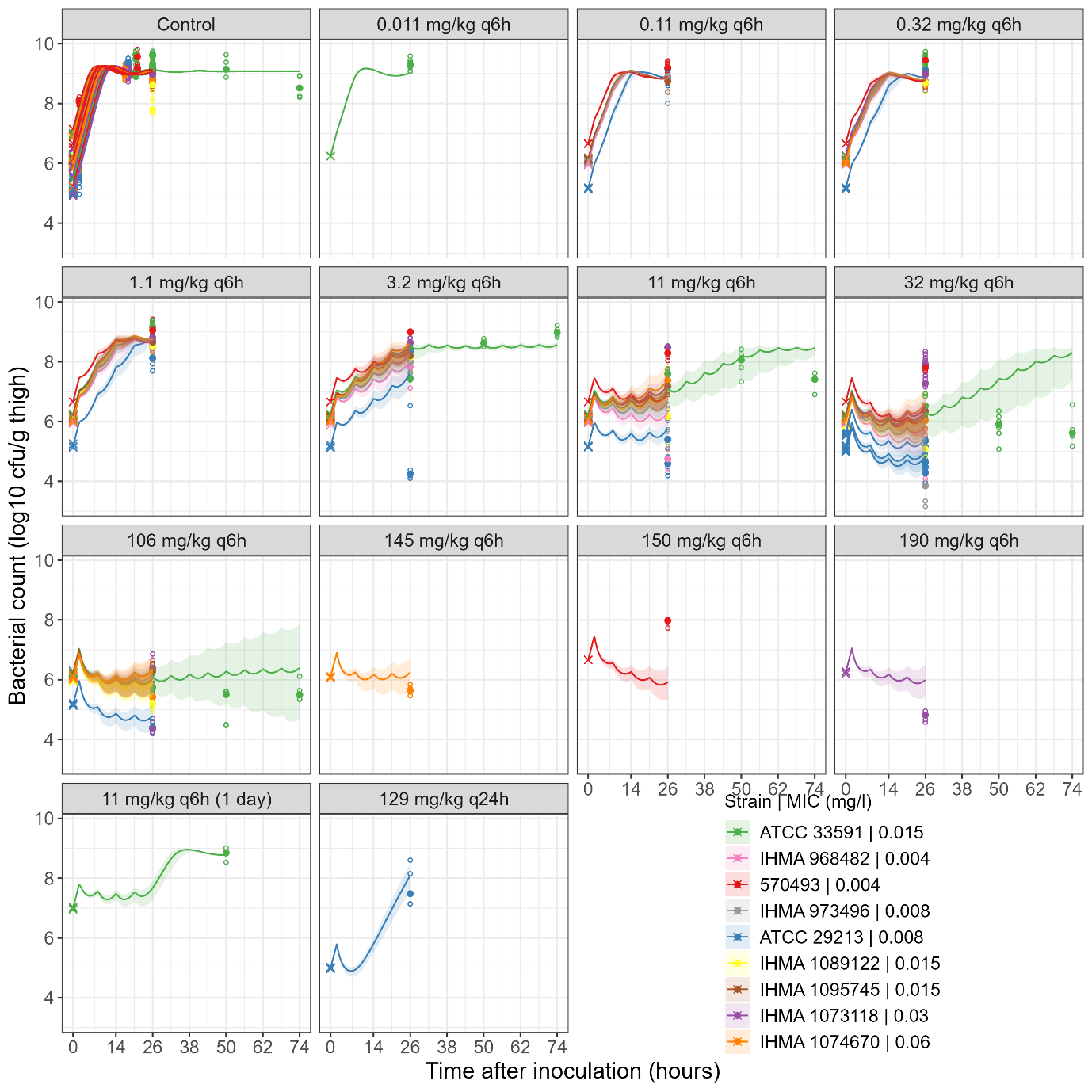


**Figure S1.** Predictions of *in vivo* efficacy based on the PKPD model for *in vitro* data, accounting for parameter uncertainty. Shown are the starting inoculum (crosses), observed bacterial counts (open circles), the median of observed bacterial counts (full circles), and the median (lines) and 95% intervals (areas) of predictions from models with parameter estimates sampled in the uncertainty distribution for the different afabicin dosing regimens and strains. Each panel presents data for all groups receiving the same afabicin regimen. Study groups with similar starting inoculum for a same strain were grouped in the plots.


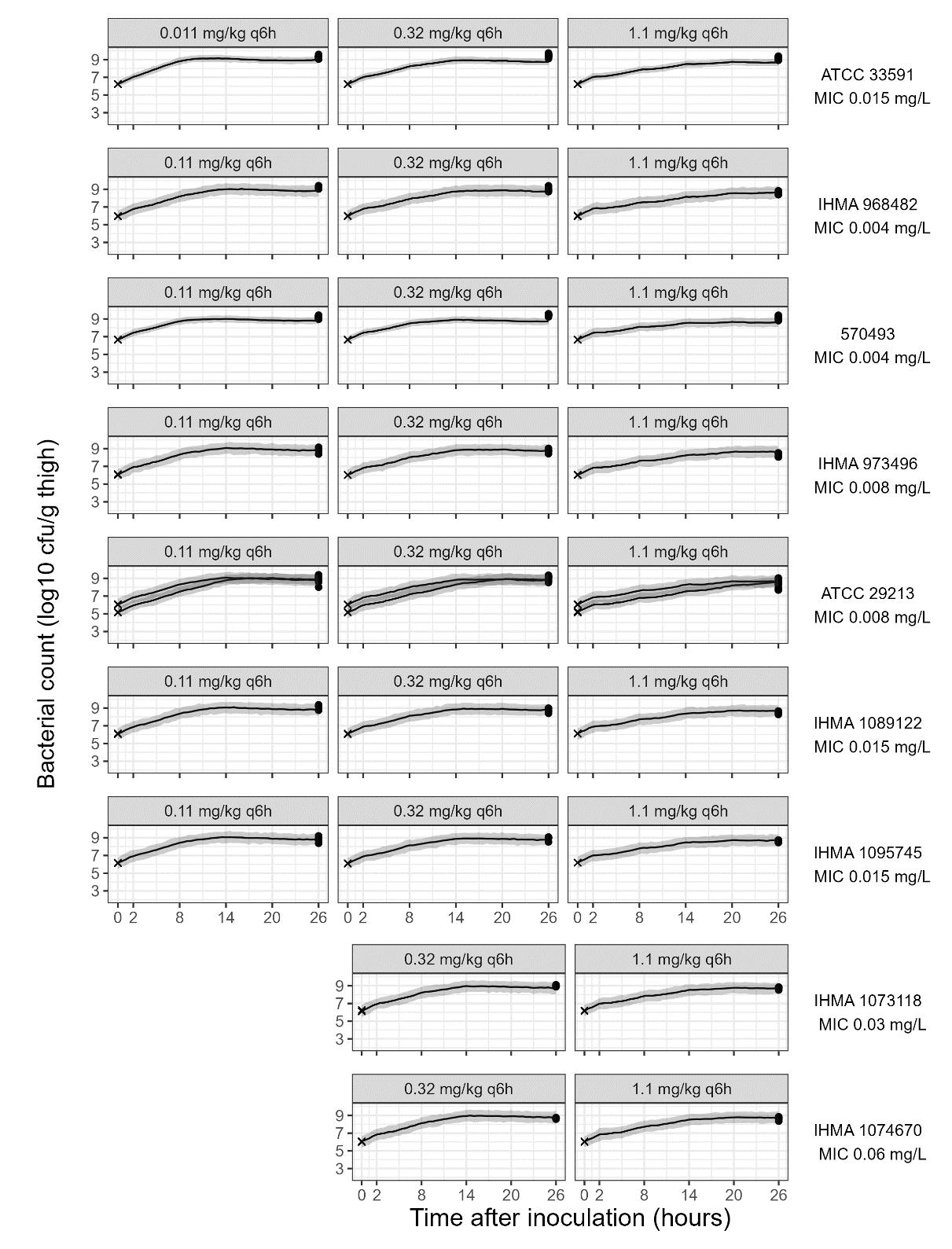


**Figure S2.** Visual predictive check of the PKPD model for *in vivo* data for afabicin doses below 3.2 mg/kg. Shown are the starting inoculum (crosses), observed bacterial counts (circles), with the median (lines) and the corresponding 95% confidence interval of the median (areas) based on model predictions for the different afabicin dosing regimen and strains. Each row presents panels containing data for the different dose groups for a given strain.
